# Biocompatible zinc battery with programmable electro-cross-linked electrolyte

**DOI:** 10.1093/nsr/nwac281

**Published:** 2022-12-14

**Authors:** Xuesong Xie, Jingjing Li, Zhengyue Xing, Bingan Lu, Shuquan Liang, Jiang Zhou

**Affiliations:** School of Materials Science and Engineering, Central South University, Changsha 410083, China; Department of Plastic Surgery, Xiangya Hospital of Central South University, Changsha 410008, China; School of Materials Science and Engineering, Central South University, Changsha 410083, China; School of Physics and Electronics, Hunan University, Changsha 410082, China; School of Materials Science and Engineering, Central South University, Changsha 410083, China; School of Materials Science and Engineering, Central South University, Changsha 410083, China

**Keywords:** polymer electrolyte, green and programmable electro-cross-linking, biocompatible zinc battery

## Abstract

Aqueous zinc batteries (ZBs) attract increasing attention for potential applications in modern wearable and implantable devices due to their safety and stability. However, challenges associated with biosafety designs and the intrinsic electrochemistry of ZBs emerge when moving to practice, especially for biomedical devices. Here, we propose a green and programmable electro-cross-linking strategy to *in situ* prepare a multi-layer hierarchical Zn–alginate polymer electrolyte (Zn–Alg) via the superionic binds between the carboxylate groups and Zn^2+^. Consequently, the Zn–Alg electrolyte provides high reversibility of 99.65% Coulombic efficiency (CE), >500 h of long-time stability and high biocompatibility (no damage to gastric and duodenal mucosa) in the body. A wire-shaped Zn/Zn–Alg/*α*-MnO_2_ full battery affords 95% capacity retention after 100 cycles at 1 A g^−1^ and good flexibility. The new strategy has three prominent advantages over the conventional methods: (i) the cross-linking process for the synthesis of electrolytes avoids the introduction of any chemical reagents or initiators; (ii) a highly reversible Zn battery is easily provided from a micrometer to large scales through automatic programmable functions; and (iii) high biocompatibility is capable of implanted and bio-integrated devices to ensure body safety.

## Teaser

A green and programmable electro-cross-linking strategy was proposed to synthesize highly hierarchical Zn–alginate electrolytes. Batteries based on this electrolyte offer potential application opportunities for *in vivo* implantation of highly demanding biocompatible devices.

## INTRODUCTION

Increasing safety concerns over the routine use of flammable and unstable organic electrolytes are challenging conventional alkali metal-ion batteries, posing a formidable barrier to the application of modernly portable and implantable electronics [1,2]. In many cases, there are potentially devastating complications associated with toxic and unstable batteries lodged in the esophagus [3], ear canal [4] and oropharynx [5], resulting in corresponding liquefactive necrosis, tissue destruction and explosively burn-related injuries [6]. Therefore, in the general survey of the strict requirements *in vivo* [7], the conviction must have been forced upon us that biocompatibility has been put forward and regarded as one of the bottlenecks. Fortunately, in achieving decently electrochemical performance in the aqueous system with neutral or mildly acidic electrolyte [8], metallic zinc (Zn)-based batteries (ZBs) have been considered as promising candidates for high safety requirements, owing to their high theoretical specific capacity (820 mAh g^−1^), low electrochemical potential (–0.762 V vs. SHE, standard hydrogen electrode), being inert in air, with low-toxicity and stability [9].

Factually, a growing increase in the literature has already demonstrated ZBs for wearable and flexible applications [10,11]. As a crucial part of the battery, the flexible hydrogel electrolyte is suitable for wearable devices to integrate with biological tissues by attaching to soft and curvilinear shapes [12], which have been developed to monitor electrophysiological signals [13], deliver therapeutic drugs [14] and be applied as a novel non-invasive therapeutic strategy for the treatment of tumors [15]. Besides the pursuit of higher electrochemical performance, numerous efforts have been dedicated to improving the flexibility and safety of the batteries themselves. An extremely tolerant and wearable solid-state ZB was fabricated by using the novel gelatin and PAM-based hierarchical polymer electrolyte [16]. The flexible ZBs successfully power the wearable pulse sensor and smart insole, and endure a variety of severe conditions. With the combination of physical and chemical cross-linking gels, the polyzwitterionic hydrogel electrolyte endows flexible ZBs with excellent processability, self-healing properties and safety [17]. Until now, multiple electrolytes have been reported for flexible ZBs, such as poly(vinyl alcohol) (PVA) [18], sodium polyacrylate hydrogel (PANa) [19], xanthan gum [20] and poly(propylene oxide)–poly(ethylene oxide) (PEO–PPO–PEO) [21]. However, in reality, the exclusive design for flexibility and the validation of the biosecurity of ZBs are still in their infancy, especially in biomedical devices, because the synthesis of the majority of polymer electrolytes was started by using noxious chemical initiators or reagents [22].

Besides the toxicity potential of electrolytes for biocompatibility, the low controllability of the synthesis will result in an uneven interfacial reaction and further aggravate the uncontrolled Zn electroplating, dendrite formation and side reaction at interfaces [23,24]. Although extensive efforts have been made in designing specific electrolytes to control the electrohydrodynamic and morphological instabilities [25], including additives modification [26], adoption of the ‘water-in-salt’ concentrated electrolytes [27–29], deep eutectic electrolytes [30–32], inorganic colloidal electrolytes [33,34] and ionic liquid electrolytes [35,36], there is a lack of suitable, green and compatible strategies to synthesize sustainable and controllable polymer electrolytes. In this case, we are inspired by the effect of the ion confinement of the carboxylate groups in nature alginate toward Zn^2+^ [37], which could become a promising polymer candidate for suppressing Zn dendrite growth and side reactions, as well as improving the biostability of natural materials. Considering that the electrodeposition method is a versatile and widely programmable method for creating metals [38], alloys [39], colloids and polymers on a conductive substrate [40], zinc metal can be used directly as an electrode because of its good electrical conductivity, chemical stability and mechanical flexibility compared with other alkali metals [41].

Herein, we developed a green electro-cross-linking method to prepare a Zn–alginate polymer electrolyte for biocompatible Zn batteries based on the superionic binding of alginate carboxylate groups with free-Zn^2+^, activated by electro-oxidation. By using this method, within a short time of 80 s, a stable polymer electrolyte with a regulated pathway (a thickness of ∼219 μm) was directly obtained on the surface of metal Zn without using noxious chemical initiators or reagents. With 5 wt% of natural nano-attapulgite additives, the ionic conductivity of the Zn–Alg-5 electrolyte successfully reached 2.5 × 10^−2^ S cm^−1^ due to the fast ion's guidance function between alginate layers. Consequently, Zn metal equipped with an ion-conductive composite polymer achieves dendrite-free and smooth Zn deposition at both low and high current densities. Meanwhile, high reversibility and stability were confirmed by the Zn plating/stripping Coulombic efficiency (CE) of 99.65% and 500 hours of long-time repeated cycling at a high current density of 5.0 mA cm^−2^. In our study, after 6 hours of exposure to the negative face of the battery cycled with the Zn–Alg-5 electrolyte, minimal mucosal injury was observed in the rabbit models, showing that the Zn–Alg-5 electrolyte is a biocompatible material that does not have any significant corrosion in the slightly acidic environment of the digestive system.

## RESULTS

**Figure 1.**
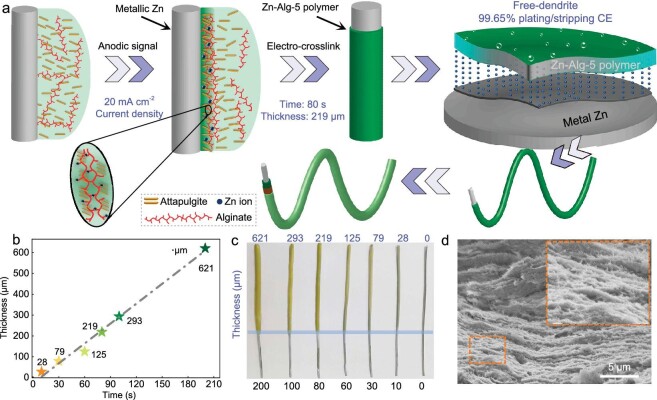
*In situ* green and programmable electro-cross-linked Zn–alginate-based polymer electrolyte (Zn–Alg-5). (a) Schematic illustration of the *in situ* preparation procedure, properties and construction of Zn–Alg-5 polymer electrolyte using a wire-shaped Zn electrode for a Zn battery. (b) The relationship between the electro-cross-linking time and the thickness of the polymer. (c) Photographs of the wire-shaped Zn after electro-cross-linking at different times. (d) SEM images of Zn-Alg-5 polymer electrolyte after electro-cross-linking.

The strategy of the electro-cross-linked preparation and application of polymer electrolytes is schematically exhibited in Fig. 1a. This process applies Zn wire as a working electrode and it was maintained for a time of 80 s at 20 mA cm^−1^ anodic current density to grow in the aqueous solution of 3.3 wt% sodium alginates and 5 wt% natural nano-attapulgite (ATP, (Mg, Al)_2_Si_4_O_10_(OH)). At the same time, the oxidized free-Zn^2+^ ions cross-link with carboxylate groups of alginate along with the introduction of ATP materials [42]. Finally, the Zn–alginate polymer electrolyte (named Zn–Alg-5 polymer) *in situ* grows on the surface of the metal Zn wire electrode and forms a film with a thickness of ∼219 μm. In the same way, the Zn–Alg-5 polymer is also *in situ* formed in planar Zn foil with the same method (Supplementary Fig. S1).

The as-prepared polymer was characterized by using Fourier-transform infrared (FTIR) spectra (Supplementary Fig. S2). The disappearance of the signals of free carboxyl groups at ∼1418 cm^−1^ (COO^−^ symmetric stretching mode) and the dramatic blue-shift of (COO^−^) asymmetric stretching from 1615 to 1628 cm^−1^ highly demonstrate the new ion bonds formation with Zn^2+^ [43], whereas the unchanged shift at ∼1615 cm^−1^ and non-cross-linked state could be found in Mg^2+^-, Na^+^- and Li^+^-ion models (Supplementary Fig. S3). X-ray diffraction patterns (Supplementary Fig. S4) demonstrate that ATP materials are present in the polymer and a clear expanded shift signal (*2θ* = 8.4°) is shown in the Zn–Alg-5 polymer compared with the pristine ATP, which may be due to the intercalation of alginate into the internal layers of the ATP [44].

Figure 1b exhibits a good linear relationship between the electro-cross-linking time and the thickness of the polymer, thus representing good programmable potential. As a consequence, the Zn–Alg-5 polymer is *in situ* covered on the wire-Zn electrode as a dense layer (Fig. 1c). The corresponding scanning electron microscope (SEM) exhibits a hierarchical structure (Fig. 1d), which is very consistent with the results of other research in terms of using a conventional chemical cross-linking method [37]. Importantly, some nano-stick ATP materials are vertically inserted into the layers (Supplementary Fig. S5), which may result in enhanced ion-guidance ability for the polymer due to its conductive carrier function [33]. After electro-cross-linking, the MnO_2_ electrode was twined onto the polymer outside to prepare the Zn/Zn–Alg-5/MnO_2_ full wire-ZBs.

**Figure 2.**
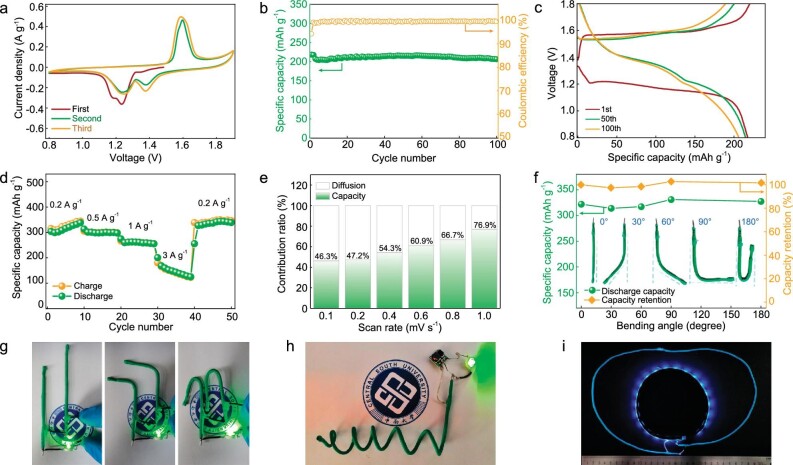
The electrochemical performance of a wire-Zn/Zn–Alg-5/MnO_2_ full battery based on this electro-cross-linking method. (a) CV curves of full wire-shaped battery at 0.1 mV s^−1^. (b) Cycling performance of full wire-shaped batteries at 1000 mA g^−1^ and (c) corresponding charge–discharge curves. (d) Rate capacity performance. (e) The capacity contribution ratios of diffusion and capacitance at different scan rates in liquid electrolyte. (f) Bending capacity and cyclic voltammetry of wire-shaped ZBs after a variety of bending angles. (g) Photographic picture of wire-shaped ZBs after bending at 90°, 0° and 180° angles. (h) Rolling wire-shaped ZBs and (i) one that is >30 cm long.

Based on this electrolyte, the electrochemical properties of wire-ZBs were further evaluated. The cyclic voltammetry (Fig. 2a, CV) shows two typical redox peaks of ∼1.25 and ∼1.38 V (vs. Zn^2+^/Zn), which are in good consistency with the traditional liquid electrolyte (2 M ZnSO_4_ + 0.2 M MnSO_4_) (Supplementary Fig. S6a). It indicates that the Zn–Alg-5 polymer-based battery possesses the same Zn storage mechanism with electrochemical Zn–insertion into *α*-MnO_2_ in a liquid electrolyte system. At a current density of 1 A g^−1^, the cell shows 95% capacity retention with a specific capacity of 206 mAh g^−1^ after 100 cycles (Fig. 2b), illustrating good electrochemical stability, which is also demonstrated in batteries with lower current densities of 0.5 and 0.2 A g^−1^ (Supplementary Fig. S6b and c). The voltage profiles of the 1st, 50th and 100th cycles are shown in Fig. 2c. The rate capability was carried out as shown in Fig. 2d. The discharge capacity is 303.6 mAh g^−1^ at a current density of 0.2 A g^−1^ and quickly recovers to 327.3 mAh g^−1^ in the same current density after the continued cycles at 0.5, 1 and 3 A g^−1^, demonstrating good rate performance and reversibility for the Zn–Alg-5 polymer. Particularly, at a high current density of 3 A g^−1^, it delivers a specific capacity of 145.2 mAh g^−1^ (on average), further confirming the high ion-diffusion capability cycled with the Zn–Alg-5 polymer. To validate the ratios of capacity contribution between the diffusion and pseudocapacitance of the Zn–Alg-5 polymer, the *b* value was calculated by using the power-law relationship of *i* =* av^b^* (*i*, peak current; *v*, scan rate; *a* and *b* are constants, *b* = 0.5 presented a diffusion-controlled capacity, whereas *b* = 1 indicates a capacitive-controlled process) [45]. The *b* values at Peaks 1, 2 and 3 are 0.63, 0.63 and 0.66, respectively, suggesting a battery-type behavior in Zn/Zn–Alg-5/MnO_2_ systems that was coincident with the liquid ones (Supplementary Fig. S7). Meanwhile, the capacitive contribution is 46.3% at 0.1 mV s^−1^ and increases to 76.9% at 1.0 mV s^−1^ (Fig. 2e), indicating the faster kinetics of ion transference enabled by the Zn–Alg-5 polymer [46].

The flexibility and expandability of these wire-shaped ZBs based on the Zn–Alg-5 polymer were also confirmed by the folding test. Figure 2f shows the changes in capability and retention under different folding angles at a current density of 200 mA g^−1^. This wire-shaped battery delivers a specific capacity of 322 mAh g^−1^ at the initial state and slightly declines at bending angles of 30^o^ and 60^o^, and then the capacity exceeds the initial one for 90^o^ and 180^o^, which may be due to the low electronic resistance (good contact of active materials) caused by the high pressure of folding. The photographic picture of wire-shaped ZBs after bending also illustrates the stability and flexibility (Fig. 2g). With this electro-cross-linking method, a large-scale ZB (>30 cm long) was made with the Zn–Alg-5 polymer as shown in Fig. 2i, proving the ease of fabrication and scalability (Fig. 2h and Supplementary Fig. S8) in the application.

The ion migration and growth mechanism of Zn deposition for the Zn–Alg-5 polymer electrolyte are analysed. Figure S9a shows the ionic conductivities of conventional liquid electrolytes, Zn–Alg-0, Zn–Alg-5 and Zn–Alg-7 polymers. Among them, Zn–Alg-0 and Zn–Alg-7 represent free and 7 wt% ATP, respectively. It should be noted that the liquid electrolyte is soaked in the glass fiber with a thickness of 431 μm. The ionic conductivities were measured by using the AC impedance of stainless steel (SS) symmetric equipment at room temperature (data listed in Supplementary Table S1). Consequently, Zn–Alg-5 exhibits the highest ionic conductivity of 2.5 × 10^−2^ S cm^−1^ and all polymer electrolytes surpass the liquid electrolyte of 1.708 × 10^−2^ S cm^−1^. Compared with other reported polymer electrolytes, Zn–Alg-5 shows highly competitive ionic conductivity (Supplementary Table S2). The reason is mostly due to the ion-guidance function of the ATP [47]. However, the lowest conductivity of ATP-7 is because the high content of the ATP material may disrupt the ordered arrangement of the gel electrolyte. The electrochemical stability was measured by using linear sweep voltammetry of Zn/Zn–Alg-5/SS cells. The electrochemical decomposition voltage of samples increases (in the order: 1.84 < 1.86 < 1.87 V vs. Zn^2+^/Zn) with the rise in ATP content from 0, 5 and 7 wt%, respectively, and all surpass the liquid system of 1.75 V (Supplementary Fig. S9b). This indicates that the polymer electrolyte is highly stable to reduce the decomposition of electrolytes.

The growth mechanism and morphology of Zn deposition were also performed by using chronoamperometry (CA). The nucleation process and surface changes are very sensitive toward the current at certain potentials [48]. When an overpotential of –200 mV is applied (Supplementary Fig. S9c), the symmetric cell cycled with the liquid electrolyte exhibits an increasing *i*–*t* (current–time) curve within 100 s, indicating a continuous and rampant 2D diffusion procedure in this system, which is commonly known as the ‘tip effect’ caused by the large electric field concentration and accumulation in the conventional electrolyte [49]. On the contrary, the 2D diffusion procedure of the Zn–Alg-5 electrolyte remains <2 s and immediately stabilizes in a constrained diffusion process with a constant current density of 5.12 mA cm^−2^, suggesting that free Zn^2+^ in the polymer electrolyte could be confined by ionic binding of carboxylate groups and lead to more uniform plating/stripping deposition (the inset of Supplementary Fig. S9c).

**Figure 3.**
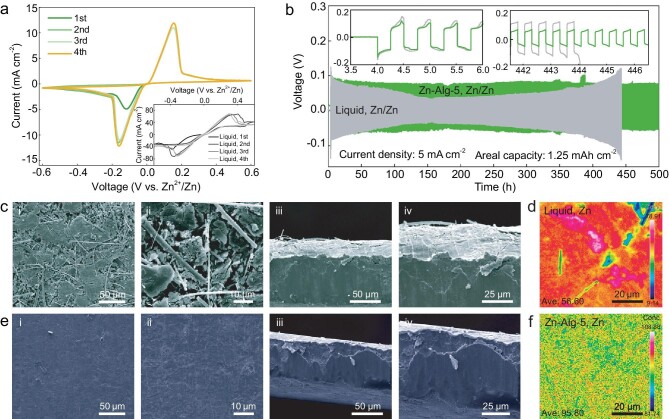
Reversibility and low level of side reactions with Zn–Alg-5 polymer electrolyte for long-time Zn plating/stripping. (a) Cyclic voltammetry (CV) curves of Zn–Alg-5 electrolyte compared with that of liquid ones at a scan rate of 2 mV s^−1^. (b) The galvanostatic cycling performance of Zn symmetric cell with Zn–Alg-5 electrolyte compared with liquid ones at a current density of 5 mA cm^−2^. The SEM images for Zn anode of the symmetrical cell after galvanostatic cycling with (c) liquid electrolyte and (e) Zn–Alg-5 polymer electrolyte at a current density of 5 mA cm^−2^ and (d and f) its corresponding EPMA/WDS mapping of Zn elements.

The Zn plating/stripping reversibility of the Zn–Alg-5 polymer electrolyte is evaluated by using Zn symmetric and Zn/carbon paper (Zn/CP) asymmetric batteries [50]. In theory, the electrochemical dissolution and deposition of Zn occur at 0 V vs. Zn^2+^/Zn, where the metal Zn electron-driven dissolution to Zn ions (Zn → Zn^2+^ + 2e^–^) at anodic current and Zn ions undergo a 2e^−^ transfer reduction reaction to metal Zn below 0 V on the contrary. For the side reactions, Zn deposition is inevitably interfered by competitive H_2_ evolution due to the low Zn^2+^/Zn potential (−0.762 V vs. SHE) and the local pH change results in insulating Zn-based by-products (such as Zn hydroxides and zincates) near the electrode surface [51], consequently leading to the limited cycling life and poor Zn utilization. As shown in Fig. 3a, CV curves of symmetry cells (Zn/Zn–Alg-5/Zn) exhibit highly symmetrical and repeatable features during the Zn plating/stripping process, suggesting the reversible dissolution and deposition of Zn. Specifically, the Zn^2+^ plating/stripping CE with the Zn–Alg-5 electrolyte is 99.65% at the end of the third cycle, which highly demonstrates that side reactions are mainly suppressed by using the Zn–Alg-5 polymer electrolyte. On the contrary, the liquid electrolyte only maintains 66.38% retention under the same measurement conditions, which also showed the same trend in CV data at a low scan rate of 1 mV s^−1^ (Supplementary Fig. S10).

To further illustrate the stability and confinement of the Zn–Alg-5 polymer electrolyte, the long-term galvanostatic cycling performance and morphology characterization for the symmetrical cell (planar Zn foil as shown in Supplementary Fig. S11a) were conducted. After 200 h of cycling at a current density of 2 mA cm^−2^, a gradual and irreversible rise in the polarization is observed in the battery cycled with the liquid electrolyte (Supplementary Fig. S11b), which stems from the accumulation of the insulating Zn-based by-products caused by irreversible reactions as mentioned above that hinder the ion transportation. The SEM images show the uneven surface and non-uniform aggregation at the Zn anode (Fig. 3c). On the contrary, Zn–Alg-5 shows a stable voltage–time curve (<0.1 V) with a small amplitude under the same conditions as well as in the low current density of 0.2 mA cm^−2^ (Supplementary Fig. S11c). The stability of the cell with the Zn–Alg-5 polymer is further verified by using SEM, which shows a clean and neat surface without dendrite formation or insulating product (Fig. 3e). The high concentration of Zn element (95.80%, Fig. 3f) for the Zn surface cycled with the Zn–Alg-5 polymer further validates the suppressed side reactions compared with the liquid electrolyte (56.60%, Fig. 3d) by using the electron probe microanalysis in wavelength-dispersive X-ray spectroscopy (EPMA/WDS, Supplementary Fig. S12). A new phase well-indexed to (Zn(OH)_2_)_3_(ZnSO_4_)(H_2_O)_3_ (JCPDS: 78–0247) caused by the parasitic precipitation of free water with OH^−^ generated by the consumption of protons due to hydrogen evolution reaction [52] was found at the Zn anode cycled with liquid electrolyte (Supplementary Fig. S13), whereas for the Zn–Alg-5 polymer electrolyte, the corresponding diffraction peaks of side reactions are almost absent. It shows good consistency with the result of CV for nearly 100% plating/stripping CE. Meanwhile, it proves that the irreversible reaction is mainly the concomitant precipitation of zinc sulfate hydroxide hydrate in the liquid electrolyte. Additionally, the stable Zn plating/stripping process for the Zn–Alg-5 electrolyte is also observed at a high current of 5 mA cm^−2^ (Fig. 3b) over 500 h with a low-level voltage hysteresis of <0.1 V (vs. Zn^2+^/Zn). In summary, with the large range of current density from 0.2 to 5 mA cm^−2^, the steady polarization and long-term durability of the battery cycled with the Zn–Alg-5 polymer highly demonstrate the depression effect of side reactions during the repeated Zn^2+^ plating/stripping process.

The suppression of side reactions for the Zn–Alg-5 electrolyte is also examined by using asymmetric Zn/carbon paper (Zn/CP) cells. Besides the stable voltage–time profile within 50 h (Supplementary Fig. S14a), the battery cycled with the Zn–Alg-5 polymer exhibits a longer discharge plateau compared with the liquid one for both the previous and last six cycles. The morphology and composition changes of the Zn surface after cycling were analysed by using SEM and EPMA/WDS. As for the liquid one, the Zn surface is rough (Supplementary Fig. S14b) and exhibits an uneven distribution of S element with a high concentration of 2.727% and a low concentration of 54.89% for Zn element (Supplementary Fig. S14d). On the contrary, the Zn surface cycled with the Zn–Alg-5 polymer is cleaner (Supplementary Fig. S14c) and displays only 0.094% concentration of S element and a high concentration and homogeneous distribution of Zn element (78.67%, Supplementary Fig. S14e). In summary, it can be demonstrated that the utility of the Zn–Alg-5 polymer notably facilitates uniform Zn deposition, avoids side reactions and guarantees reversibility during cycling.

**Figure 4.**
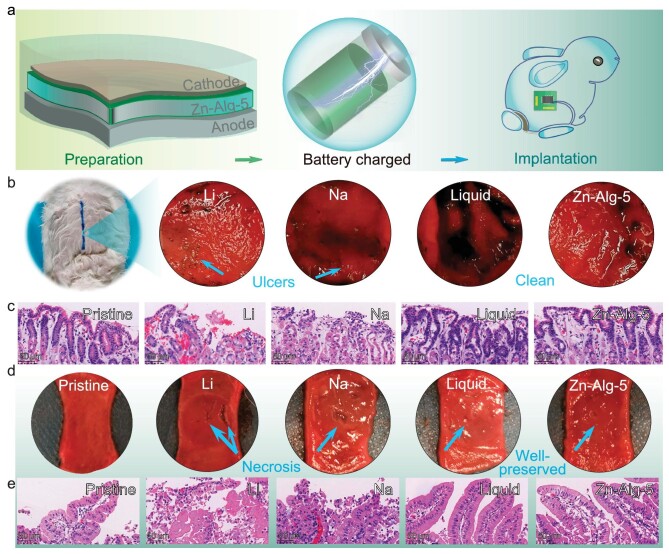
Biocompatibility and safety studies of batteries implanted in the rabbit. (a) Schematic diagram of biocompatible applications using rabbit models. (b) Photographic picture of gastric walls and (c) the HE-stained section of its mucosa after the undamaged batteries were implanted in the stomach for 6 hours. (d) Pictures of freshly extracted duodenum tissue and (e) the HE-stained section of its mucosa after the punched batteries were implanted in the duodenum organ for 6 hours.

The biocompatibility and safety of this battery with the Zn–Alg-5 polymer were conducted using rabbit models (Fig. 4a). To better compare the benign nature of the aqueous-based electrolyte and this Zn–Alg-5 polymer electrolyte prepared by using the green electro-cross-linking method, the research also assembled typical lithium- and sodium-ion batteries with organic electrolytes (1 M NaPF_6_ with EC : DEC = 1 : 1 solution, 1 M LiPF_6_ with EC : DEC : DMC = 1 : 1 : 1 solution). The gastric mucosa is the innermost layer of the stomach wall. As shown in Fig. 4b, the battery was placed on the gastric mucosa for 6 hours to simulate the effects on organic tissue when the battery is directly exposed to the body. The gastric tissue was evaluated by gross and histological analysis. Grossly, both lithium- and sodium-ion batteries-treated gastric mucosa developed surface ulcers, whereas Zn–Alg-5- or liquid-treated gastric mucosa did not experience this complication. Assessment of the hematoxylin-eosin (HE)-stained sections showed full-thickness necrosis of the mucous layer and extensive infiltration of neutrophils, characterized by caustic injury of organic solvent due to electrolyte leakage [53]. On the contrary, for the batteries cycled with liquid and Zn–Alg-5 polymer electrolytes, structural integrity, mucous secretions as well as lack of damage were observed in the gastric mucosa (Fig. 4c).

Three mechanisms have been proposed for the batteries-related damage: being placed on the mucous membrane resulting in pressure-induced local ischemia, leakage of electrolytes causing chemical corrosion and electrical current created by contact of the battery to the mucous membrane. It is worth noting that batteries are able to cause damage even under discharge conditions, implying that battery contents are thought to cause the most damage, especially in implant devices. The electrolytes in aqueous media exhibit better biocompatibility than highly corrosive organic electrolytes. This can be further confirmed by the results obtained by drilling a hole in the cell with a 1-mm diameter needle to initiate electrolyte leakage (Supplementary Fig. S15), where severe gastric mucosal ulceration and tissue congestion can be observed in lithium- and sodium-ion batteries, whereas there is a lack of mucosa damage in batteries cycled with liquid and Zn–Alg-5 electrolyte.

Additionally, the perforated batteries were placed on the freshly extracted duodenum for 6 hours to evaluate the biocompatibility. As shown in Fig. 4d, a lack of intestinal mucosa damage observed in the duodenum treated with the Zn–Alg-5 electrolyte-based battery has the smallest damaged area, which may be due to the inherently biocompatible alginate materials [54]. The HE-stained section of its duodenum mucosa (Fig. 4e) also confirms structural integrity without necrosis of the intestinal villus after treatment with the Zn–Alg-5 electrolyte-based battery. In contrast, the cupped lesions were observed in the duodenum tissues after treatment with the lithium and sodium cells. The HE-stained sections showed extensive liquefaction necrosis without any recognizable structure in the duodenum tissues after treatment with the lithium and sodium cells. In summary, the battery cycled with the Zn–Alg-5 polymer electrolyte has benign biocompatibility for the stomach and duodenum organs in rabbits, which is a good opportunity for the application of implantable devices to ensure the body's safety even under the damaged circumstances compared with typical lithium- and sodium-ion batteries.

## CONCLUSION

In summary, we have successfully developed a versatile programmable and green electro-cross-linking method to prepare a durable and flexible polymer-state electrolyte based on Zn ions that can cross-link alginate. The superior ionic binding towards carboxylate groups of alginate leads to the formation of stable layer-by-layer hierarchical networks. Based on the flexible structure, the wire-shaped Zn/Zn–Alg-5/MnO_2_ full battery delivers outstanding electrochemical performance, high stability and flexibility. Validated by the biosafety study in lab rabbits, the strategy and merit of polymer electrolyte provide an opportunity for the design of future biosafety Zn batteries in need of eye-catching modern portable and wearable devices as well as implanted devices. Meanwhile, it significantly expands green, programmable and sustainable strategies for the preparation of polymer electrolytes.

## Supplementary Material

nwac281_Supplemental_File
