# Supplementary material for: Biocompatible zinc battery with programmable electro-cross-linked electrolyte
Source: Natl Sci Rev. 2022 Dec 14;10(3):nwac281. doi: 10.1093/nsr/nwac281 (PMC9976762; doi:10.1093/nsr/nwac281)
Supplement: nwac281_Supplemental_File [file nwac281_supplemental_file.doc]

**Supporting Information for**

**Biocompatible Zinc Battery with Programmable Electro-Crosslinked Electrolyte**

*Xuesong Xie^1^, Jingjing Li^2^, Zhengyue Xing^1^, Bingan Lu^3^, Shuquan Liang^1^ and Jiang Zhou^*1^*

*^1^School of Materials Science and Engineering, Central South University, Changsha, 410083, P.R. China;^2^Department of Plastic Surgery, Xiangya Hospital of Central South University, Changsha, 410008, P.R. China;^3^School of Physics and Electronics, Hunan University, Changsha 410082, P. R. China. E-mail address: zhou_jiang@csu.edu.cn*

X.X. and J.L. contributed equally to this work.

**Experimental Section**

**Synthesis of Zn-Alg-5 polymer electrolyte.** All the reagents are of analytical purity and used as received without further purification. It takes Zn wire (φ = 0.959 mm, 5 cm length) as a working electrode, and remains 80 s time at a constant 20 mA cm^-1^ anodic current density in the aqueous solution of 3.3 wt% sodium alginates and 5 wt% attapulgites at room temperature. The copper foam (2 × 8 cm) was adopted as the counter electrode. After electrodeposition, the polymer Zn-Alg-5 polymer was successfully prepared, where the Alg-0, and Alg-7 represented the ratio of 0 wt%, 7 wt% attapulgite materials. The *α*-MnO_2_ was prepared by the hydrothermal method. 0.2 g MnSO_4_·H_2_O and 0.5 g KMnO_4_ were dissolved in 20 ml deionized water and stirred, respectively. Then, two solutions were mixed at room temperature for 1 h. After that, the solution was transferred to a Teflon-lined autoclave (50 mL) and heated at 160 ^o^C for 12 h. After cooling, the precipitation was collected by centrifugation, washed three times with deionized water, and dried in an air oven at 70 ^o^C.

**Assembly of wire-shaped full batteries.** The positive MnO_2_ material, conductive carbon black, and PVDF binder (polyvinylidene difluoride) are dissolved in organic solvent NMP (1-Methyl-2-pyrrolidinone), mixed evenly with a mass ratio of 7:2:1 and scraped onto the stainless steel foil collector, dried at 60 ^o^C for use. The electrode sheet is 1 mm*50 mm rectangular in size, as the positive electrode. To avoid the Mn dissolution, the polymer electrolyte was also immersed in a 0.2 M MnSO_4_ solution for 12 h and dried at room temperature. Then, the MnO_2_ electrode can be assembled into a full cell by winding it around the integrated negative electrode (wire-Zn electrode with in-situ electrolyte). Finally, the full cell was sealed by a heat-sealed tube to obtain the wire-shaped Zn/MnO_2_ full batteries.

**Characterizations.** Powder X-ray diffraction (XRD) patterns were conducted by the Rigaku Mini Flex 600 diffractometer using Cu K_α_-radiation (*λ*= 1.5418) with a scan speed of 2°/min. XPS measurements were carried out on an ESCALAB 250 Xi X-ray photoelectron spectrometer (Thermo Fisher). Morphology images were collected on a FESEM (FEI Nova NanoSEM 230, 10kV) field emission scanning electron microscope. Electron probe microanalysis was performed on a JXA-8230 instrument with wavelength-dispersive X-ray spectroscopy (WDS) characterization. The high/low-temperature performance was conducted by using a high/low-temperature test box (LAND GT2001B, China).

**Electrochemical Measurements.** The bare Zn foil was punched into disks (*Φ* = 15 mm) and served as the planar pristine Zn anode (Nantong Xianxiang Zinc Industry Co., Ltd.). The cathode and anode for the liquid cell were separated by glass fiber separators (*Φ* = 19 mm, Whatman) and 2 M ZnSO_4_ + 0.2 M MnSO_4_ aqueous solution was used as the typical electrolyte. The cells were assembled in the air atmosphere. Galvanostatic charge/discharge cycling measurements were carried out on a LAND multichannel battery test system (CT2001A, China). CV data was recorded on the electrochemical workstation (CHI660E, China). Linear sweep voltammetry (LSV) was carried out at a scan rate of 50 mV s^-1^ where stainless steel was used as the counter electrode and Zn foil as the working electrode. The galvanostatic intermittent titration technique (GITT) was tested in the Arbin instruments system at room temperature.

**Biocompatibility and safety research**. This study was approved by the Department of laboratory animals of Central South University (CSU-2022-0122). New Zealand White Rabbits weighing 2.5 to 3.2 kg were randomly distributed into four experimental groups and were anesthetized to a surgical plane of depth using pentobarbital sodium (30–45 mg/kg intravenous [IV]). Select the midline abdominal incisions to free the whole stomach. Open the stomach along the greater curvature and clean the stomach instantly. The stomach wall is divided into four regions. A battery (battery without electrolyte leakage or battery with electrolyte leakage after punching with 1 mm diameter) was placed on each region to allow the cathode to make contact with the gastric mucosa surface. After 6 hours, the batteries were removed, and the tissue injury was evaluated by gross and histologic analysis. The fresh extracted duodenum tissue was used to validate the biocompatibility of batteries further, and the protocol was performed according to the stomach injury models. The research type is homologous control, that is, one animal is given four kinds of battery stimulation at the same time, excluding the influence of individual differences of animals on the experimental results, repeated 3 times.

**Supplementary Materials**


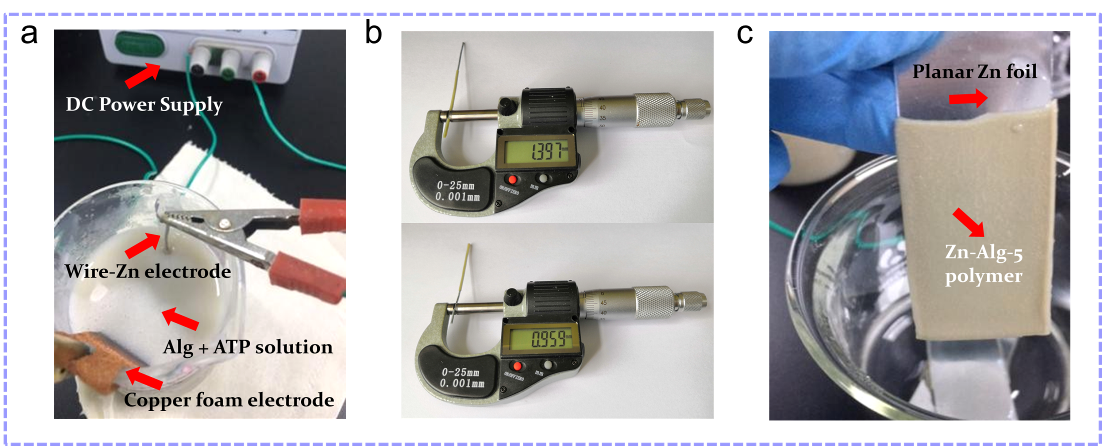


**Fig. S1. The electro-crossing devices.** (**a**) The electro-crosslinking components and photographic images of the polymer electrolyte using the wire-Zn as the working electrode. (**b**) The corresponding result of Zn-Alg-5 polymer. (**c**) Planar-Zn foil working electrodes.


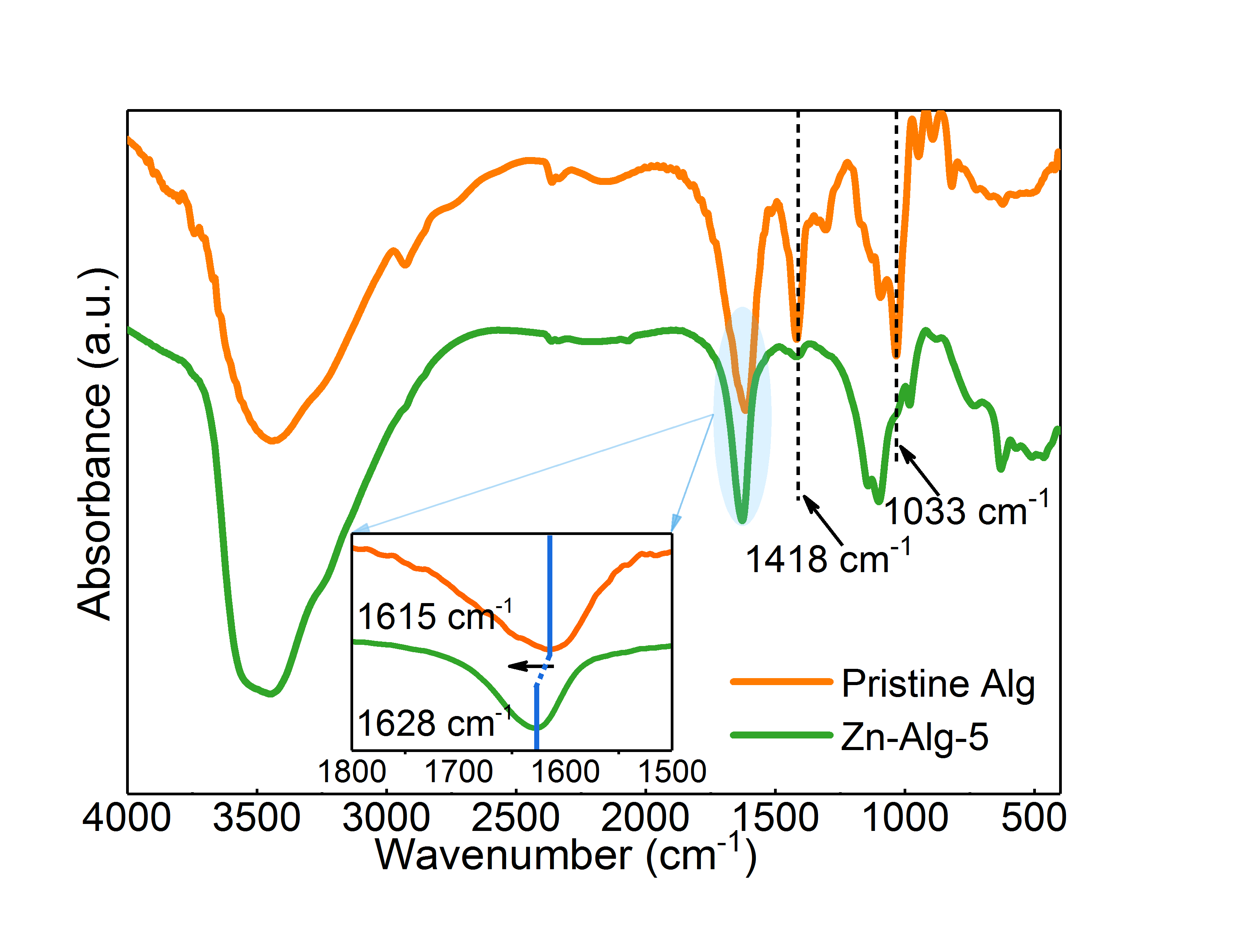


**Fig. S2. FTIR spectra of pristine Alg and Zn-Alg-5 polymer in the range of 400 ~ 4000 cm^-1^.**


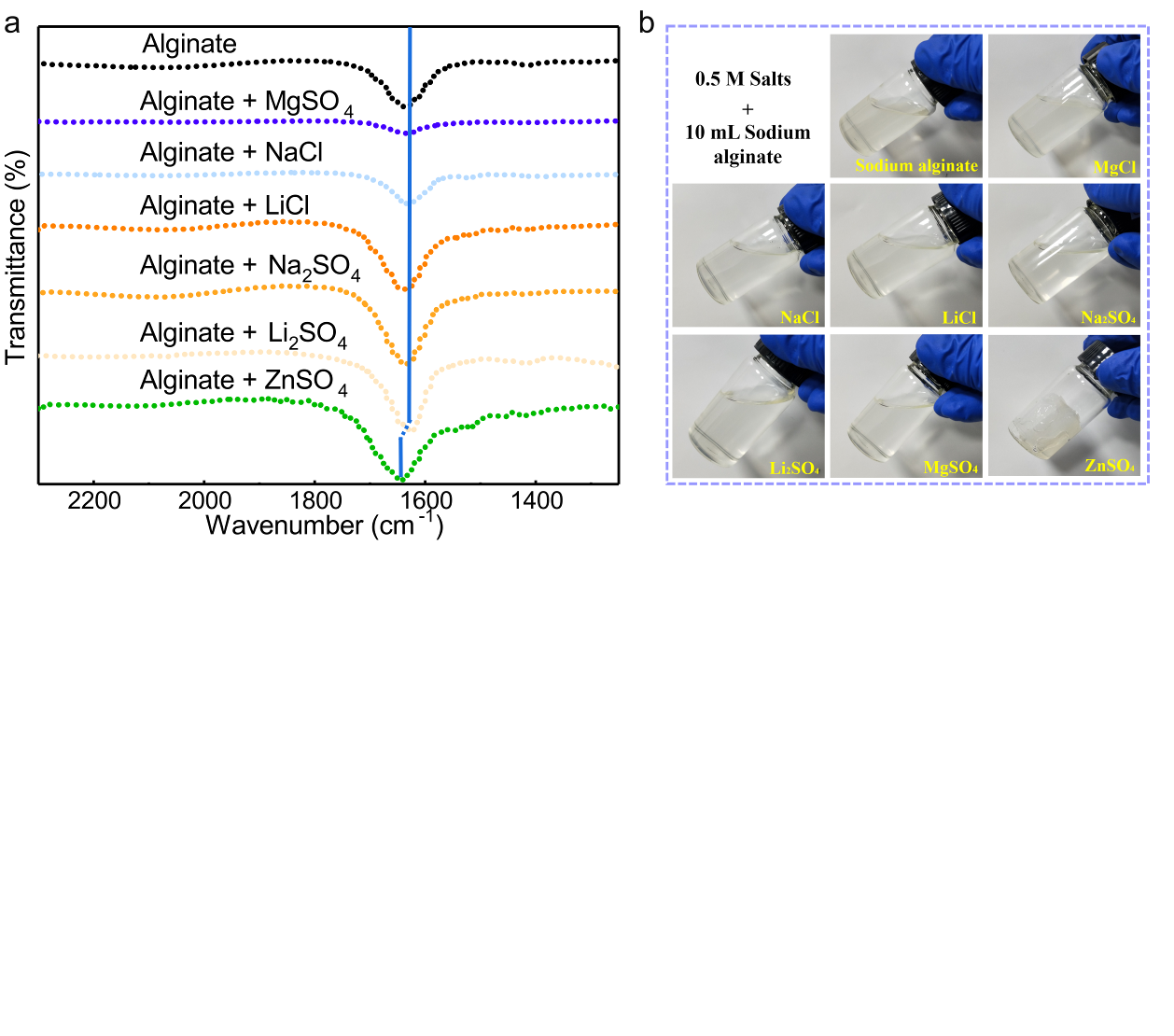


**Fig. S3. FTIR spectra and photographic images.** (**a**) FTIR spectra of alginate with different salts in the range of 1300 ~ 2300 cm^-1^. (**b**) Corresponding photographic images of sodium alginate with different salts, respectively.


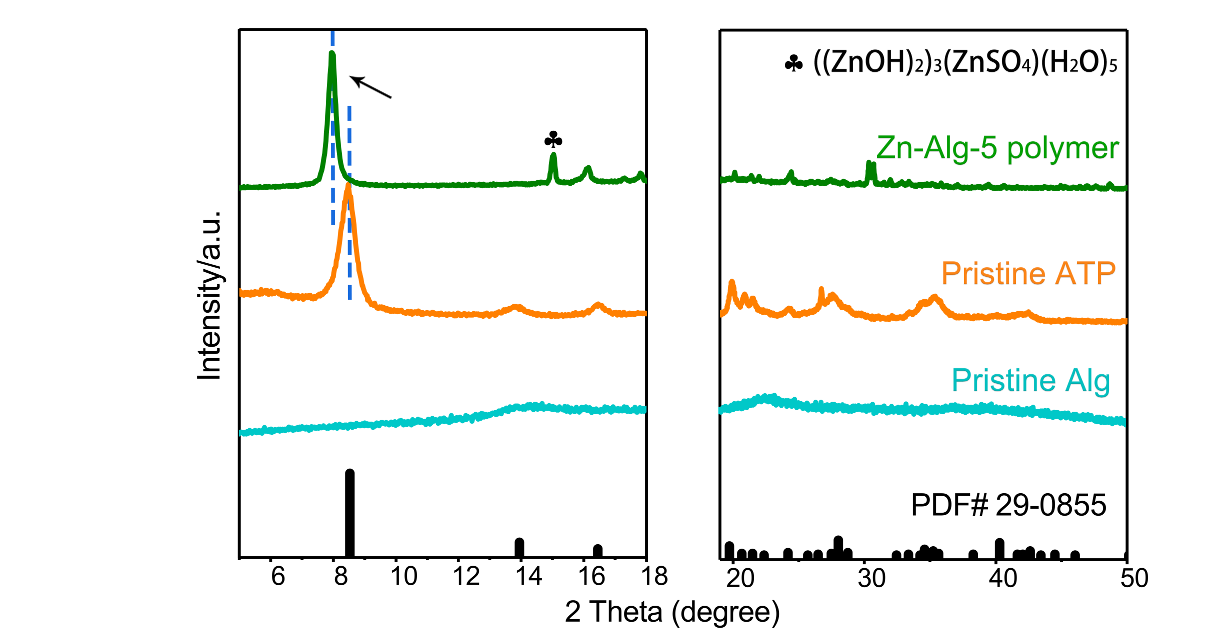


**Fig. S4. XRD spectra of the pristine alginate and Zn-Alg-5 polymer.**


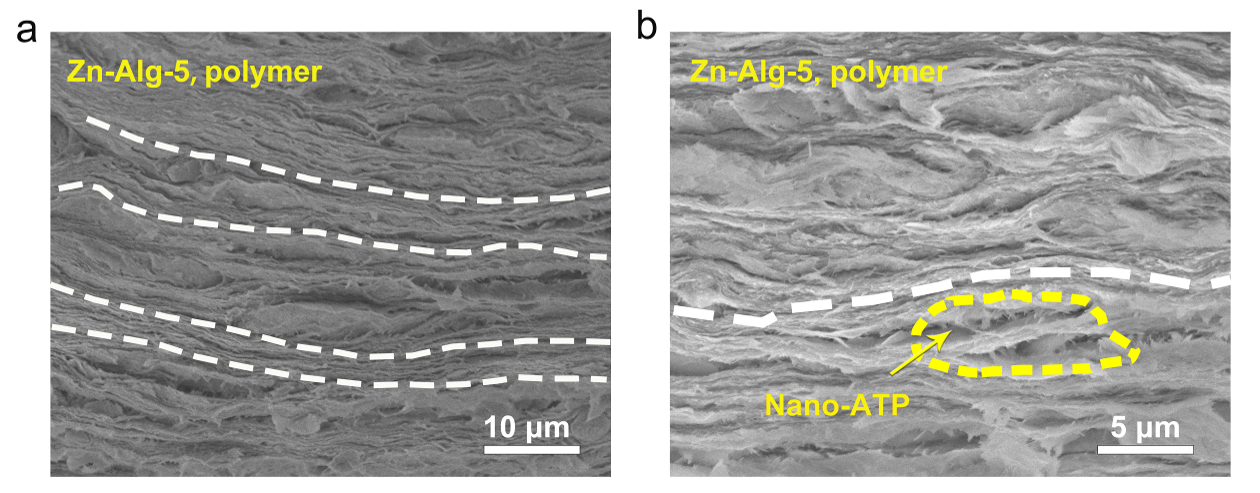


**Fig. S5. SEM figures of Zn-Alg-5 polymer electrolyte after drying.**


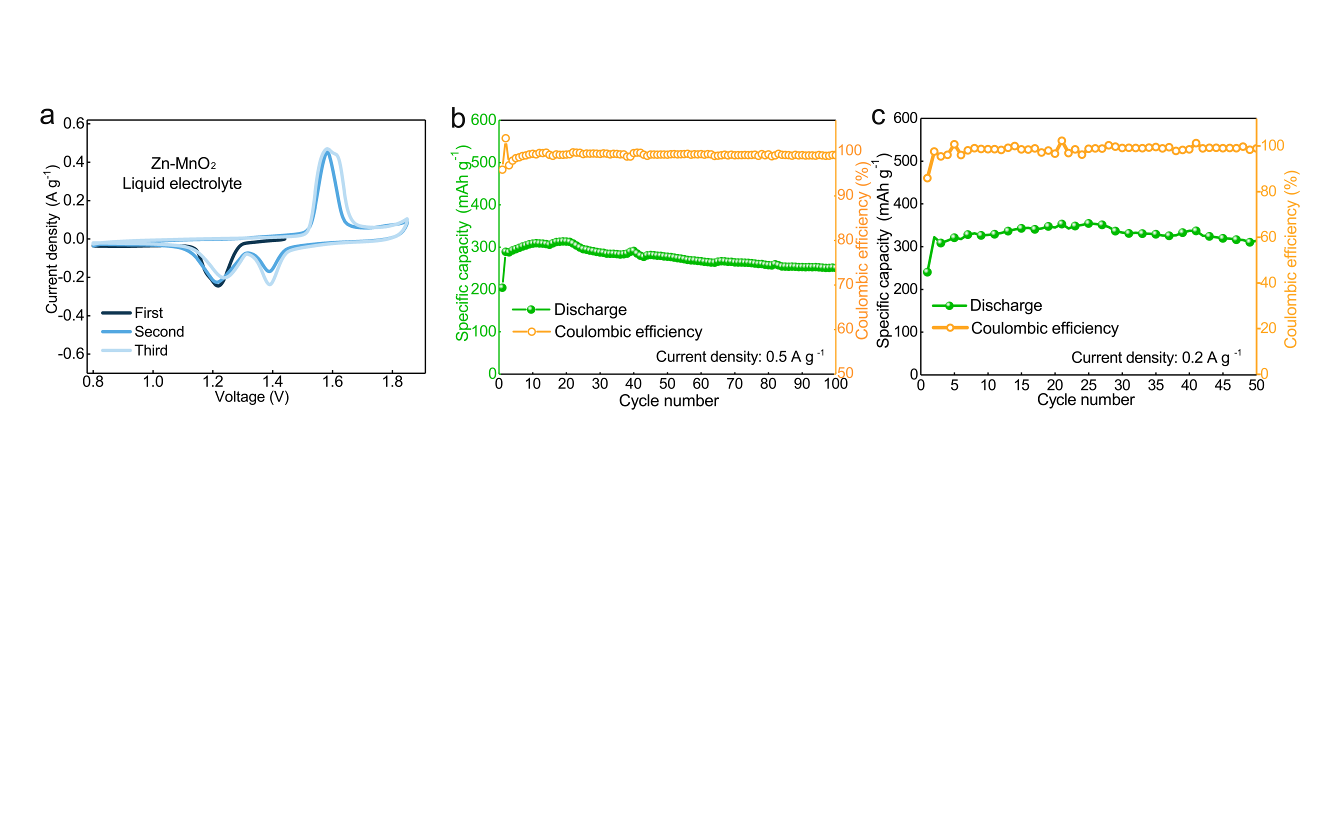


**Fig. S6.** (**a**) CV curves of Zn/MnO_2_ battery cycled with normal liquid electrolyte. Cycling performance of full wire-shaped batteries at 0.5 A g^-1^ (**b**) and 0.2 mA g^-1^ (**c**), respectively.


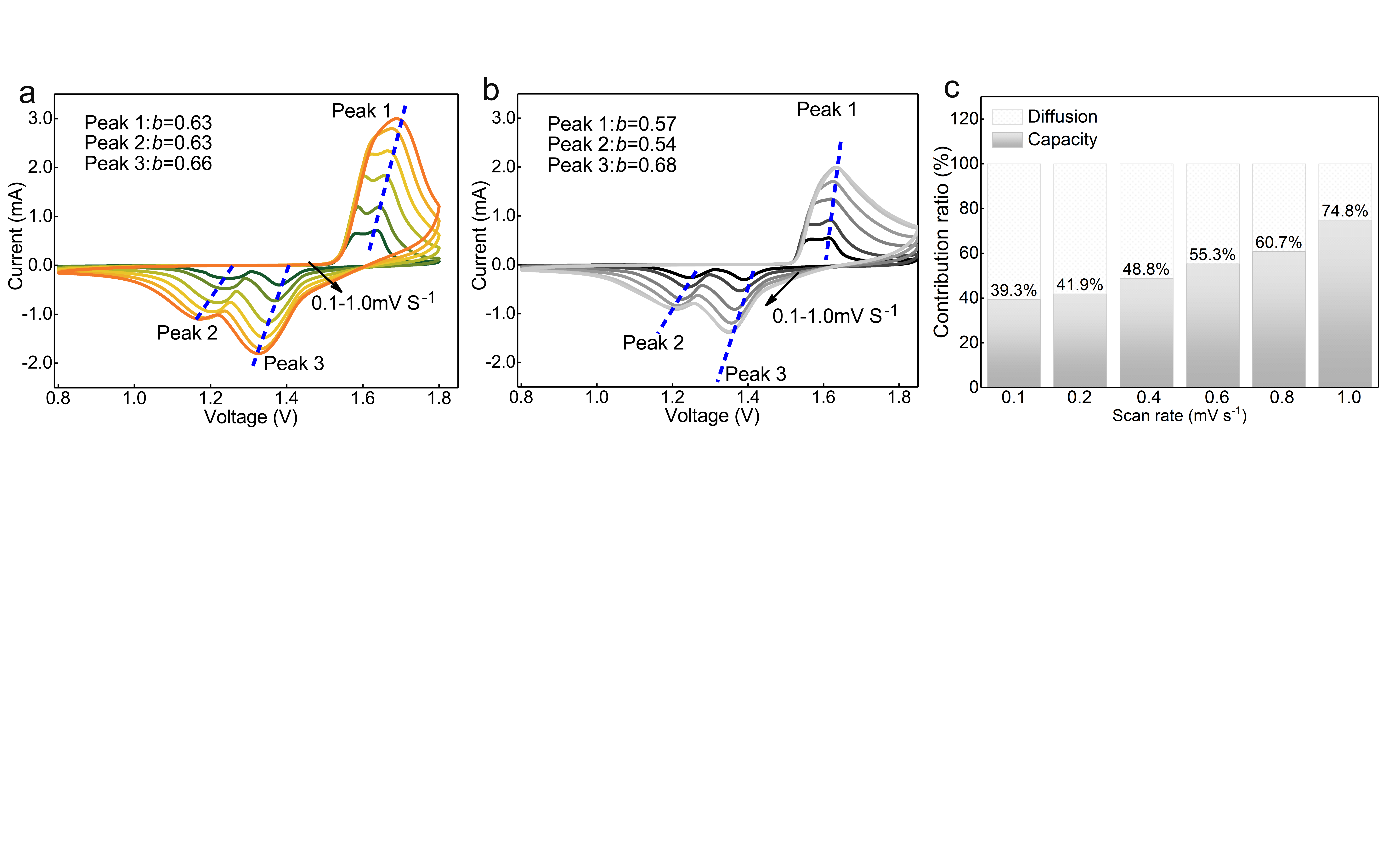


**Fig. S7.** Cyclic voltammetry (CV) curves at different scan rates for MnO_2_ cells cycled with liquid (**a**) and Zn-Alg-5 polymer electrolytes (**b**), respectively. (**c**) Corresponding capacitance and diffusion contributions of cells cycled with liquid electrolyte.

**
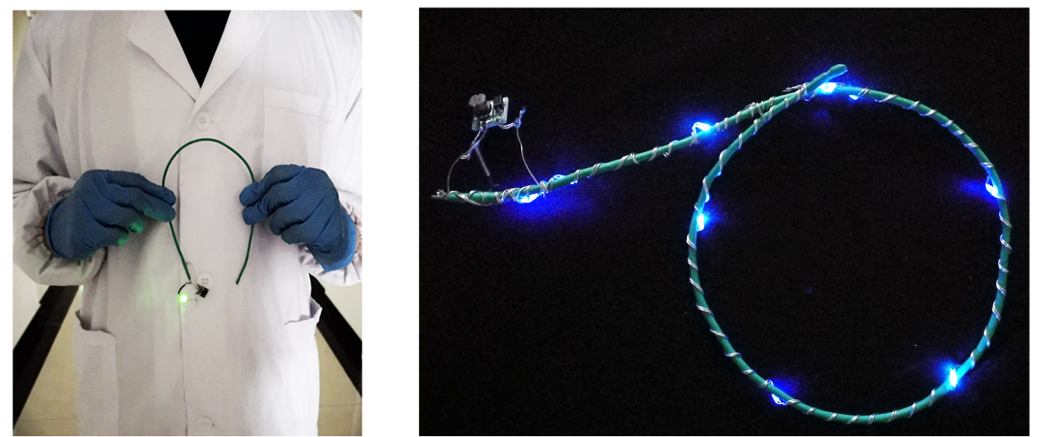
**

**Fig. S8.** Physical photos of more than 30 cm long linear batteries.


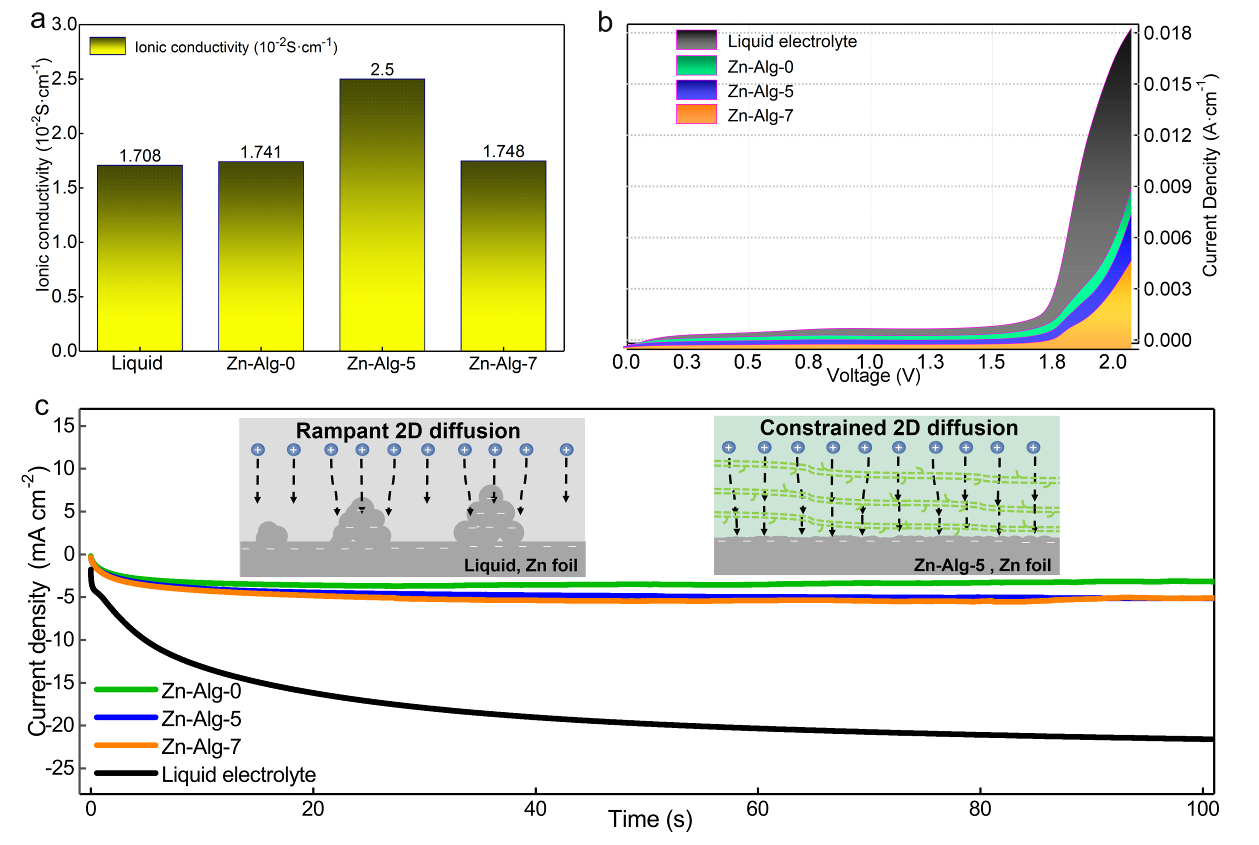


**Fig. S9. Properties of Zn-Alg-5 polymer electrolyte.** (**a**) Ion conductivity and (**b**) linear scan voltammetry (LSV) curves of the as-prepared electrolyte (with different contents) and a liquid one. (**c**) Chronoamperometry (CA) curves of as-prepared electrolyte and liquid one at a -200 mV overpotential.


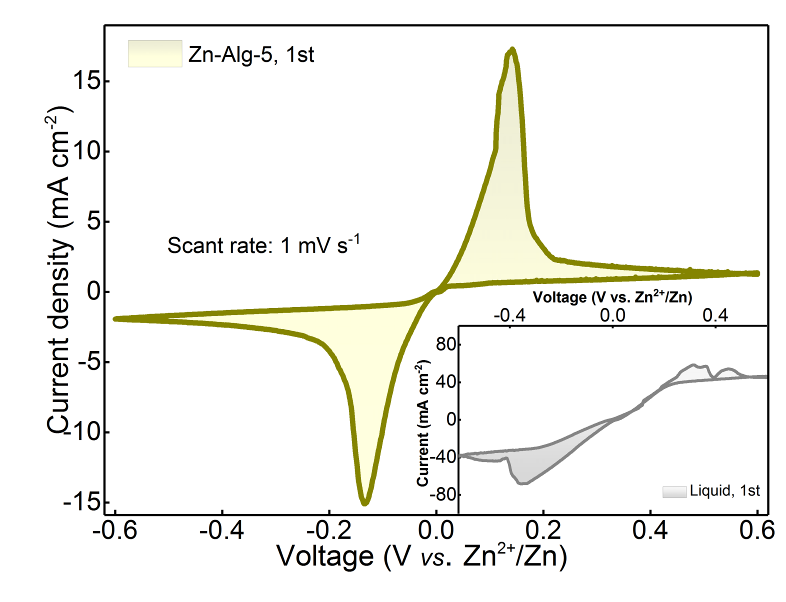


**Fig. S10.** Cyclic voltammetry curves of Zn-Alg-5 electrolyte compared to that of liquid ones at 1 mV s^-1^.


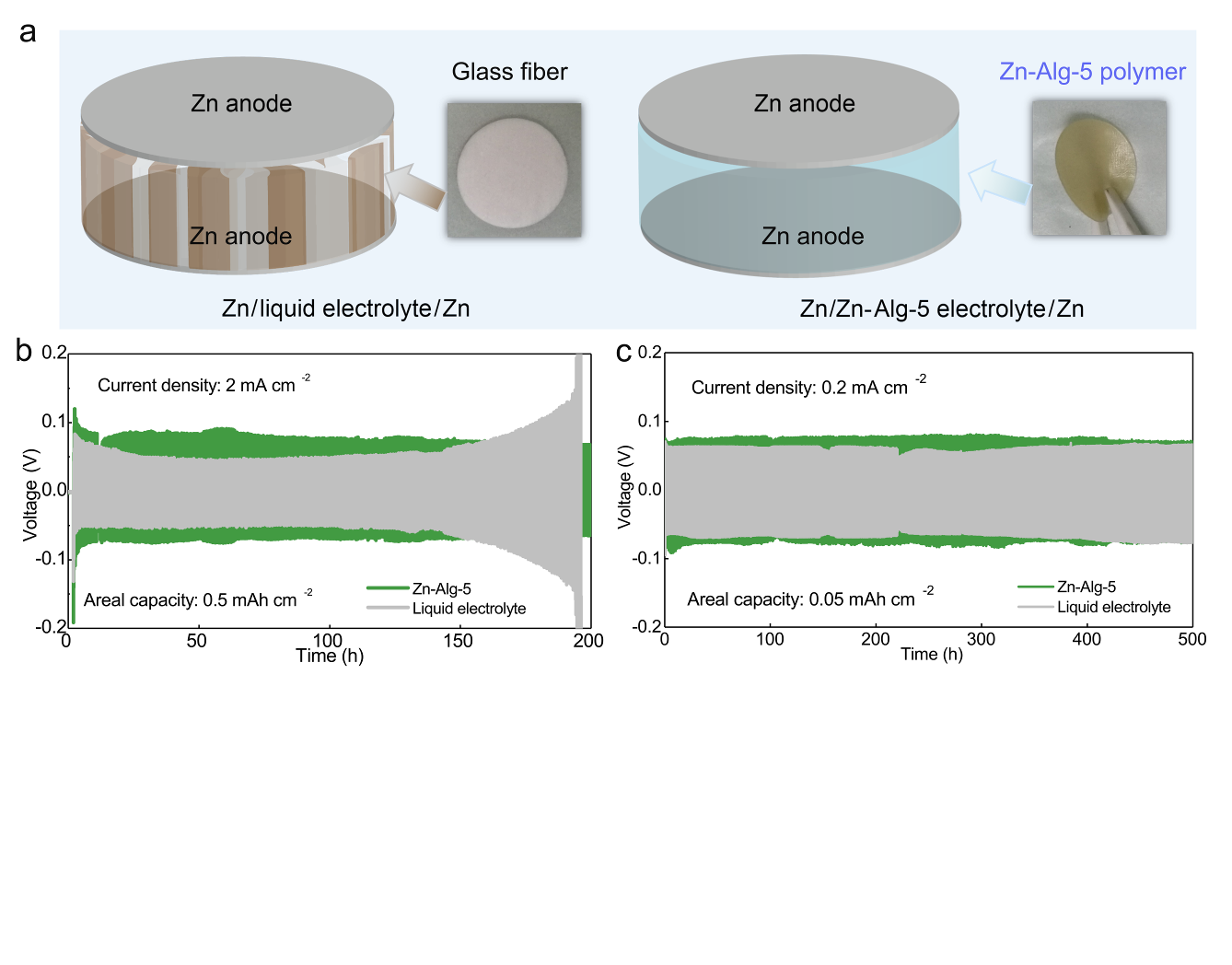


**Fig. S11.** (**a**) The schema and photographic figures of symmetric Zn/Zn cell with liquid and Zn-Alg-5 polymer electrolytes, respectively. Galvanostatic cycling performance of Zn symmetric cell with Zn-Alg-5 electrolyte and liquid ones (glass fiber as separator with 2 M ZnSO_4_ + 0.2 M MnSO_4_ liquid electrolyte) at a current density of 2 mA cm^-2^ (**b**) and 0.2 mA cm^-2^ (**c**), respectively.


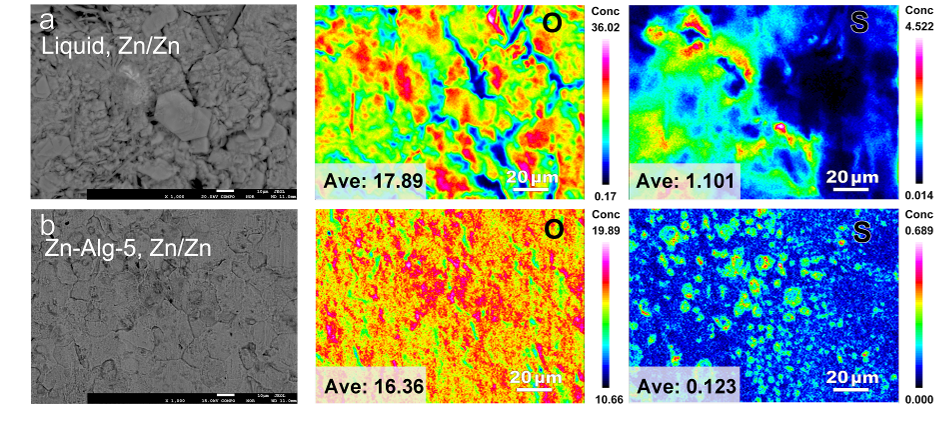


**Fig. S12.** The EPMA/WDS images of Zn anode after cycled with liquid electrolyte (**a**) and Zn-Alg-5 polymer (**b**) in a symmetrical battery for O, and S elements distribution, respectively.

**Fig. S13.** The XRD patterns of Zn anodes before and after 200 cycles of galvanostatic cycling for symmetric cells at 2 mA cm^-2^ current density with Zn-Alg-5 electrolyte and liquid one, respectively.


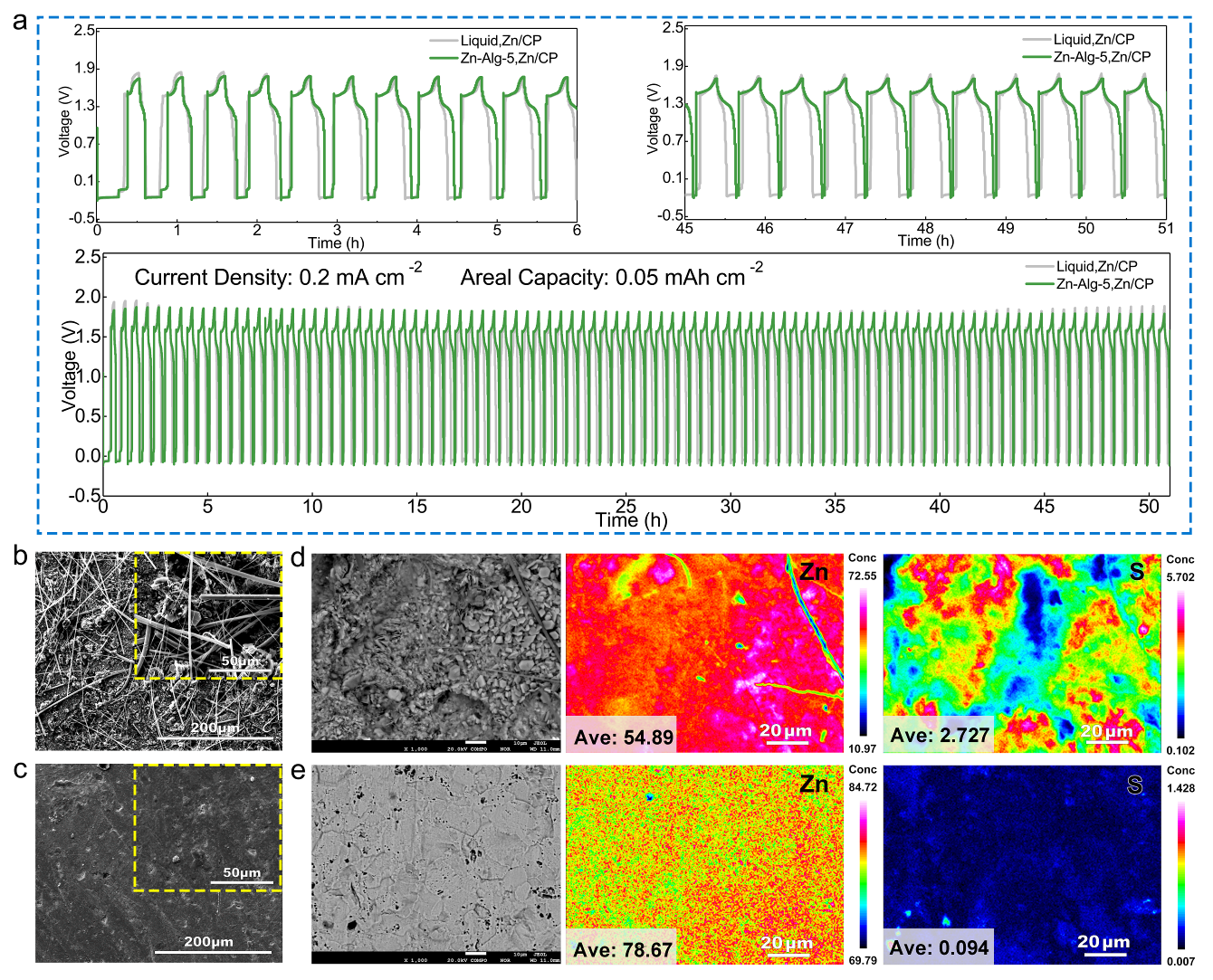


**Fig. S14. The electrochemical performance and surface characterizations of Zn/carbon paper (Zn/CP) asymmetric cells with Zn-Alg-5 electrolyte and liquid ones.** The galvanostatic cycling performance of Zn/CP cell at a current density of 0.2 mA cm^-2^ for the before and after 6 hours as well as the entire cycling (**a**). The SEM images for Zn anode after galvanostatic cycling of the asymmetrical cell with liquid electrolyte (**b**) and polymer electrolyte Zn-Alg-5 (**c**). EPMA/WDS images of Zn anode after cycled with liquid electrolyte (**d**) and Zn-Alg-5 polymer (**e**) for Zn, S elements.


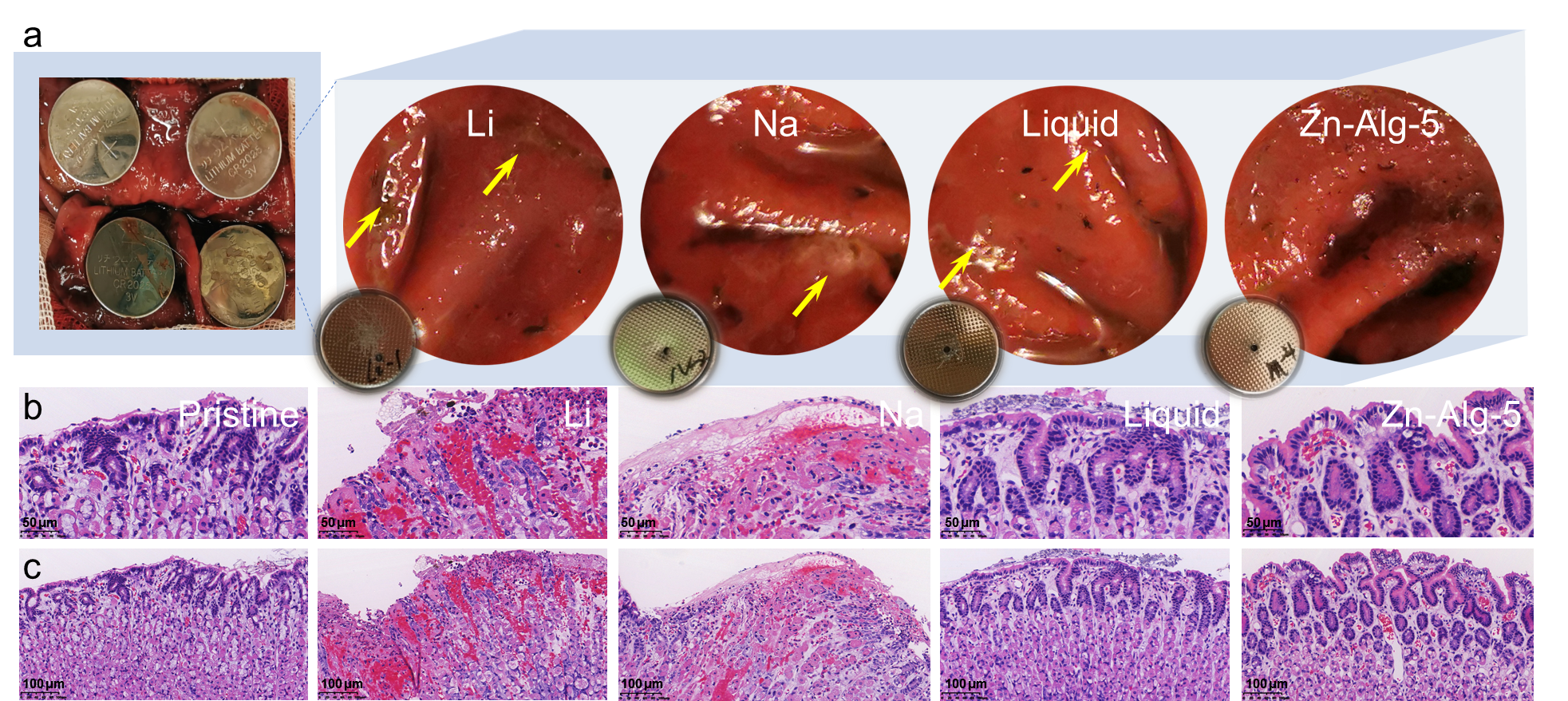


**Fig. S15. The biocompatibility and safety studies of batteries implanted in the rabbit.** The picture of gastric wall (**a**) and the HE-stained section of the gastric mucosa (**b** and **c**) after the punched batteries were implanted in the stomach for 6 hours. For the lithium and sodium ion batteries-treated tissue, the section of gastric mucosa showed extensive hemorrhage and necrosis with abundant neutrophil infiltrations. For the liquid electrolyte-treated tissue, gastric mucosa was integral and cell structures were clear with a small hemorrhagic focus. For the Zn-Alg-5 electrolyte-treated tissue, lack of mucosa damage was observed under the microscope.

**Table S1**. Ionic conductivity of both as-prepared electrolyte and liquid ones (glass fiber, 431μm thickness).

| Separator | liquid | Zn-Alg-0 | Zn-Alg-5 | Zn-Alg-7 |
| --- | --- | --- | --- | --- |
| Bulk resistance  (Ω) | 1.287 | 1.263 | 0.879 | 1.257 |
| Ionic conductivity  (10^-2^ S cm^-1^) | 1.708 | 1.741 | 2.5 | 1.748 |

As a result, the ionic conductivity is measured by AC impedance of steel/electrolyte/steel symmetry equipment at room temperature as followed in Eq. (1)

$\sigma\text{=}\frac{l}{R\text{·}S}$ (1)

Where *l* is the thickness of the gel electrolyte, *R* is the bulk resistance according to the EIS measurement, and *S* is the contact area of the electrolyte.

**Table S2.** Comparison of the ionic conductivities of polymer electrolytes.

| Polymer matrices | Salts | | Conductivity  (S cm^-1^) | | References | | |
| --- | --- | --- | --- | --- | --- | --- | --- |
| Zn-Alg-5 | | 2 M ZnSO_4_ + 0.2 M MnSO_4_ | | 2.5 × 10^-2^ | | ***This work*** |  |
| PAM-5%Lap | | 2 M ZnSO_4_ + 0.2 M MnSO_4_ | | 2.07 × 10^-2^ | | *ACS Appl. Mater. Interfaces* **2022**, 14, 25962-25971 |  |
| PAAm/agr | | 1 M Zn(CF_3_SO_3_)_2_ | | 1.55 × 10^-2^ | | *ACS Appl. Mater. Interfaces* **2022**, 14, 23452-23464 |  |
| Agarose/PAM/CMC | | 1 M ZnSO_4_ + 0.2 M MnSO_4_ | | 2.31 × 10 | | *Mater. Today Phys.* **2021**, 20, 100458 |  |
| Cellulose/TEOS/glycerol | | 0.45 M ZnSO_4_ + 0.045 M MnSO_4_ | | 3.23 × 10^-2^ | | *Adv. Mater.* **2021**, 33, 2007559 |  |
| GG/SA/EG | | 2 M ZnSO_4_ + 0.1 M MnSO_4_ | | 1.681 × 10^-3^ | | *Energy Storage Mater.* **2021**, 41, 599-605 |  |
| PMAEDS | | 2 M ZnSO_4_ + 0.1 M MnSO_4_ | | 2.46 × 10^-2^ | | *Adv. Energy Mater.* **2020**, 2000035 |  |
| Polyzwitterionic hydrogel | | 2 M ZnSO_4_ | | 3.2 × 10^-2^ | | *Adv. Funct. Mater.* **2020**, 30, 2001317 |  |
| Starch-PAM hybrid | | 2 M ZnSO_4_ | | 2.65 × 10^-2^ | | *Energy Storage Mater.* **2019**, 19, 94-101 |  |
| PVA/glycerol | | 2 M ZnSO_4_ + 0.2 M MnSO_4_ | | 1.0 × 10^-2^ | | *J. Mater. Chem. A* **2020**, 8, 6828 |  |
| Silane-modified Zn-alginate/PAM | | 2 M ZnSO_4_ + 0.1 M MnSO_4_ | | 1.63 × 10^-2^ | | *EcoMat.* **2019**, 1, e12008. |  |
| PAM | | 2 M ZnSO_4_ + 0.1 M MnSO_4_ | | 1.73 × 10^-2^ | | *ACS Nano*  **2018**, 12, 3140-3148 |  |
| Cellulose/PAM | | 2 M ZnSO_4_ + 0.2 M MnSO_4_ | | 2.28 × 10^-2^ | | *Small*  **2018**, 14,1803978 |  |
| Gelatin and PAM | | 2 M ZnSO_4_ + 0.1 M MnSO_4_ | | 1.76 × 10^-2^ | | *Energy Environ. Sci.*  **2018**, 11, 941-951 |  |
| Xanthan gum | | 3 M ZnSO_4_ + 0.1 M MnSO_4_ | | 1.65 × 10^-2^ | | *J. Mater. Chem. A* **2018**, 6, 12237-12243 |  |
| PVA | | 3 M LiCl + 2 M ZnCl_2_ + 0.4 M MnSO_4_ | | 0.897 × 10^-2^ | | *Adv. Mater.*  **2017**, 29, 1700274 |  |
| Poly-ε-caprolactone | | 2 M Zn(CF_3_SO_3_)_2_ | | 8.8 × 10^-4^ | | *Express Polym. Lett.*  **2013**, 7, 495 |  |
